# Effect of music therapy on children with autism spectrum disorders in the Chinese population: a systematic review and meta-analysis

**DOI:** 10.3389/fpsyt.2025.1611182

**Published:** 2025-07-25

**Authors:** Yajing Yang, Yu Wang, Wenhui Li, Li Su

**Affiliations:** ^1^ School of Film, Xiamen University, Xiamen, China; ^2^ Health Management Center, Xiang’an Hospital of Xiamen University, Xiamen, China; ^3^ School of Art, Xiamen University, Xiamen, China

**Keywords:** music therapy, children, autism spectrum, meta-analysis, ATEC, CARS

## Abstract

**Objectives:**

This study aimed to investigate the efficacy of music therapy (MT) in children with autism spectrum disorder (ASD) in the Chinese population through a meta-analysis.

**Design:**

Systematic review and meta-analysis.

**Setting:**

We performed a systematic review according to the PRISMA guidelines. A systematic and comprehensive search was conducted across five databases: PubMed, Web of Science, Chinese Knowledge Infrastructure (CNKI), China Science and Technology Journal Database, and Wanfang Database, up to the end of September 2024, that administered MT to children with ASD. Continuous variables were reported as standardized mean differences (SMD) accompanied by 95% confidence intervals (CIs). All analyses were carried out using the Stata statistical software package version 16.0.

**Results:**

Twenty-three studies were included in our meta-analysis. The results showed that MT significantly decreased the total ATEC (autism treatment evaluation checklist) score (SMD = -2.52, 95% CI: -3.69 ~ -1.35, P < 0.001), ABC (autism behavior checklist) (SMD = -1.07, 95% CI: -1.52 ~ -0.61, P < 0.001), and CARS (childhood autism rating scale) score (SMD = -1.50, 95% CI: -2.26 ~ -0.74, P < 0.001). Specifically, MT significantly improved communication skills (SMD = -1.10, 95% CI: -1.54 ~ -0.66, P < 0.001), social interaction skills (SMD = -1.69, 95% CI: -2.59 ~ -0.78, P < 0.001), language ability (SMD = -1.15, 95% CI: -1.56 ~ -0.74, P < 0.001), and cognitive function (SMD = -1.80, 95% CI: -2.73 ~ -0.87, P < 0.001) compared to the control group.

**Conclusion:**

MT can improve communication skills, social interaction skills, language ability, and cognitive function in children with ASD disorders in the Chinese population.

## Introduction

Autism Spectrum Disorder (ASD), commonly referred to as autism, is a neurodevelopmental disorder that typically appears before the age of three and is characterized by challenges in social communication, as well as repetitive behaviors and restricted interests (1, 2). ASD includes a variety of disorders with different causes and symptoms, characterized by significant challenges in social interaction and communication, a broad spectrum of severity, and the potential for co-occurring issues such as intellectual disabilities, anxiety, depression, sleep disturbances, epilepsy, and exceptional talents in specific areas, often referred to as savant abilities (3).

Epidemiological surveys reveal a global median prevalence of ASD at 62 per 10,000 individuals, with the 2010 Global Burden of Disease study estimating approximately 52 million people affected worldwide, translating to a prevalence of about 1 in 132 (4). In China, the Second Epidemiological Sample Survey of the Disabled identified around 41,000 children aged 0 to 6 with ASD (5). Furthermore, a recent comprehensive study involving over 120,000 children aged 6 to 12 from eight representative cities in China reported an estimated prevalence of 0.70% (6). The significant increase in ASD prevalence observed over the past two decades raises serious public health concerns.

Music therapy (MT) is a cost-effective and noninvasive adjunct to standard treatment, demonstrating effectiveness in addressing psychiatric disorders across a variety of settings and patient demographics, and it is generally easy to implement (7). Moreover, MT has been shown to enhance cognitive functions such as attention and memory (8). Notably, many children with autism, despite their limited engagement with the external world and occasional language challenges, often exhibit a keen interest in music and may even possess exceptional musical perception and superior sound discrimination skills (9). This unique connection makes MT particularly prominent among interventions for autism.

Therefore, this meta-analysis was conducted to assess the effects of MT on children with ASD in the Chinese population.

## Materials and methods

This systematic review adheres to the Preferred Reporting Items for Systematic Reviews and Meta-Analyses (PRISMA) guidelines for reporting and has been prospectively registered in the PROSPERO database under registration number CRD42015029643.

### Search strategy and selection criteria

In accordance with the Cochrane Collaboration guidelines, a systematic and comprehensive search was conducted across five databases: PubMed, Web of Science, Chinese Knowledge Infrastructure (CNKI), China Science and Technology Journal Database, and Wanfang Database, up to the end of September 2024. The PubMed search combined the terms (“music therapy”[Title/Abstract] OR MT[Title/Abstract]) AND (“autism”[Title/Abstract] OR “autism spectrum disorder”[Title/Abstract]) AND (“children”[Title/Abstract] OR child*[Title/Abstract]) AND (“autism treatment evaluation checklist”[Title/Abstract] OR ATEC (autism treatment evaluation checklist) [Title/Abstract] OR “autism behavior checklist”[Title/Abstract] OR ABC (autism behavior checklist) [Title/Abstract] OR “childhood autism rating scale”[Title/Abstract] OR CARS (childhood autism rating scale) [Title/Abstract]). In Web of Science we applied the analogous query TS = (“music therapy” OR MT) AND TS= (autism OR “autism spectrum disorder”) AND TS = (children OR child*) AND TS = (“autism treatment evaluation checklist” OR ATEC OR “autism behavior checklist” OR ABC OR “childhood autism rating scale” OR CARS). Equivalent strategies using the same concepts translated into Chinese were applied in CNKI, the China Science and Technology Journal Database and the Wanfang Database. Initially, all articles published in English or Chinese were considered for inclusion in the analysis.

### Inclusion and exclusion criteria

Inclusion criteria: (1) Studies published in peer-reviewed journals investigated the effects of MT delivered by credentialed therapists following established therapeutic protocols on children with ASD; (2) specifically measuring at least on of outcomes using ATEC, ABC, or CARS; (3) Only articles published in English or Chinese and conducted up to September 2024 will be considered. Exclusion criteria: (1) Studies that are not empirical research, including reviews, meta-analyses, case reports or case series; (2) Studies focused on populations other than children with ASD or that do not specifically address MT as an intervention will also be excluded; (3) studies involving non-human subjects.

### Data extraction

Duplicate publications were removed using EndNote 21 software, and manual searching of grey literature (e.g., conference abstracts) to reduce publication bias. Two authors independently extracted the data using standardized forms and resolved any discrepancies through discussion until a consensus was reached. Any disagreements were resolved through discussion; if needed, a third author arbitrated. The extracted information included the following: author names, publication year, study location, sample size, age, sex, intervention methods, and duration of intervention.

### Quality assessment

The quality of the studies included in this review was assessed by two independent reviewers using the Newcastle-Ottawa Scale (NOS) (10), which evaluates three key parameters: selection, comparability, and exposure, resulting in a score ranging from 0 to 9. Studies received classifications based on their scores, with those scoring 0 to 3 deemed low quality, scores of 4 to 6 categorized as moderate quality, and scores of 7 or higher recognized as high quality. To ensure the integrity of the findings, only studies with NOS scores of 6 or above were included in the analyses, thereby enhancing the reliability of the review’s conclusions and ensuring that they are based on high-quality evidence.

### Statistical analysis

All analyses were carried out using the Stata statistical software package version 16.0. Continuous variables were reported as standardized mean differences (SMD) accompanied by 95% confidence intervals (CIs). To assess heterogeneity, either the Chi-square test or the Cochrane Q test was employed, with I² < 50% and P > 0.10 indicating no significant heterogeneity. A fixed-effects model was used when these criteria were satisfied; otherwise, a random-effects model was applied. Furthermore, funnel plots along with the Egger test were utilized to investigate publication bias, where a p-value of < 0.05 was taken as evidence of statistically significant publication bias.

## Results

### General results of the included studies

**Figure 1:**
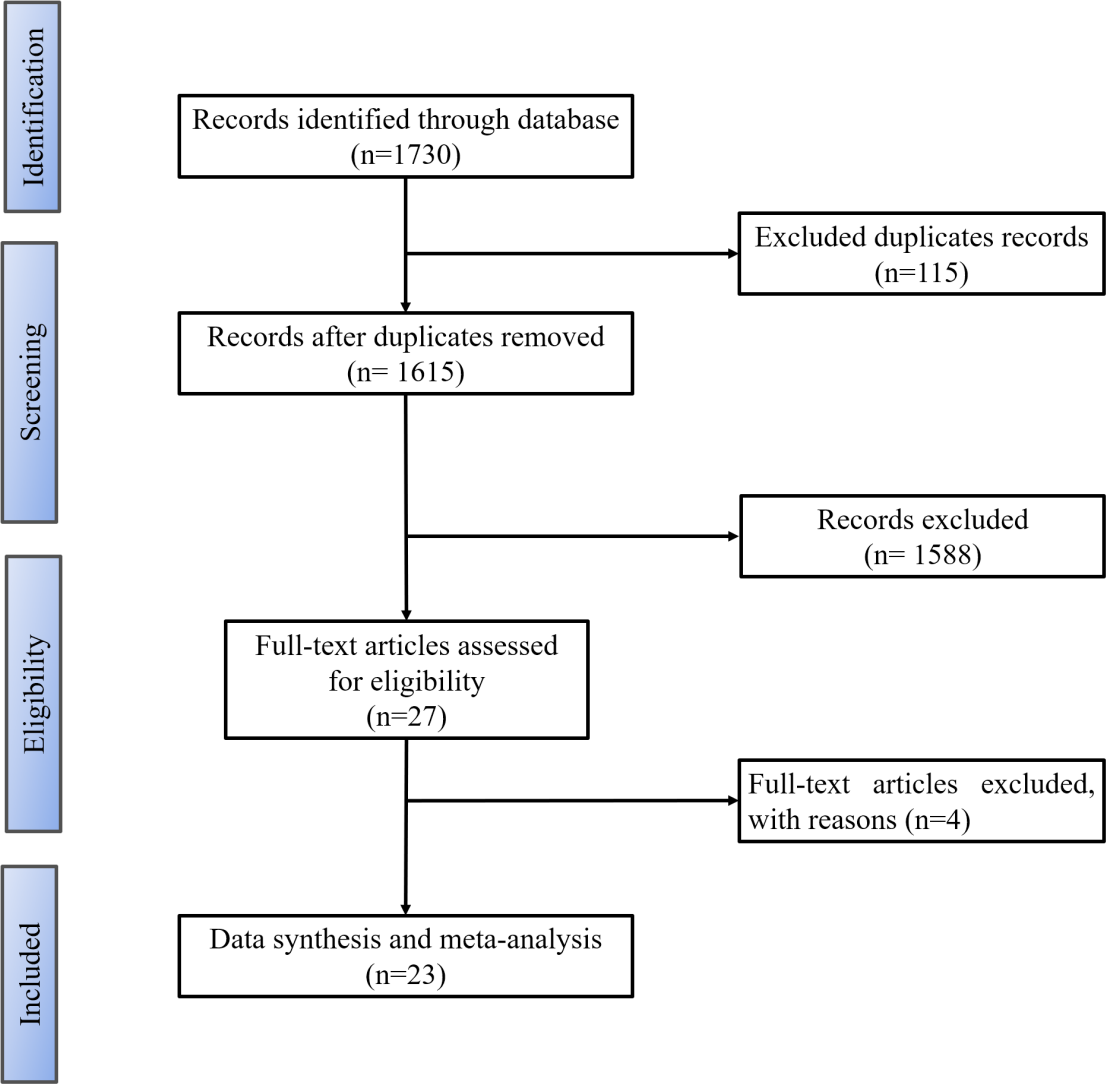
PRISMA flow chart. Showing the article selection process.

Following the specified inclusion and exclusion criteria, a total of 1,730 articles were initially obtained. After eliminating 115 duplicates and disregarding 950 articles classified as irrelevant, an additional 177 articles were excluded upon full-text assessment. Finally, 27 articles were chosen for qualitative analysis, with 23 retained for meta-analysis (**Figure 1**).

### Characteristics of included studies

The cohort of the studies that evaluated the included articles comprised a total of 1,684 participants, with 840 individuals in the observation group and 844 individuals in the control group. The quality assessment of the 23 selected articles was conducted using the NOS scale. Among these, 20 articles received a NOS score of 8, while the remaining 3 articles scored 7. This indicates that the overall quality of the articles included in this study is relatively high (**Table 1**).

**Table 1:** Characteristics of included studies.

Authors	Year	Sample size		Age		Sex(M/F)		Intervention measure		Duration (week)	NOS
		MT	Ctrl	MT	Ctrl	MT	Ctrl	MT	Ctrl		
Duan et al. (11)	2019	45	45	5.30 ± 2.00	5.20 ± 2.20	33/12	32/13	MT (IMT) + language training	Language training	12	8
Wu et al. (12)	2019	33	32	4.51 ± 0.72	4.48 ± 0.70	29/4	29/3	MT (Receptive methods) + general rehabilitation training	General rehabilitation training	24	8
Sui et al. (13)	2017	35	35	5.41 ± 1.77	5.36 ± 1.75	19/16	21/14	MT (Mixed methods) + analytical therapy	Analytical therapy	12	8
Zhou et al. (14)	2017	53	55	3.79 ± 1.12	3.72 ± 1.08	40/13	43/12	MT (IMT) + routine rehabilitation training	Routine rehabilitation training	24	8
Fu et al. (15)	2016	45	45	4.50 ± 0.50	3.50 ± 0.50	22/23	20/25	MT (Receptive methods) + language training	Language training	24	8
Li et al. (16)	2016	40	40	3.32 ± 1.23	3.45 ± 1.30	27/13	31/9	MT (Mixed methods) + medical education	Medical Education	10	8
He et al. (17)	2016	30	30	4.20 ± 0.30	4.30 ± 0.20	25/5	26/4	MT (Community MT) + routine training	Routine training	12	8
Sun et al. (18)	2014	22	21	4.02 ± 3.14	3.78 ± 4.05	19/3	19/2	MT (Receptive methods) + acupuncture therapy	Acupuncture therapy	12	8
Zhang et al. (19)	2014	40	40	4.23 ± 1.10	4.38 ± 1.05	28/12	30/10	MT (Receptive methods) + routine training	Routine training	12	8
Liu et al. (20)	2011	30	40	NA	NA	27/3	33/7	MT (Receptive methods) + language cognitive training	Language cognitive training	10	8
Wang et al. (21)	2009	26	26	4.20 ± 5.68	4.20 ± 5.68	8/18	8/18	MT (Receptive methods) + routine basic care	Routine basic care	24	8
Liu et al. (22)	2006	29	28	5.89 ± 2.08	5.93 ± 2.46	26/3	24/4	MT (Receptive methods) + integrative treatment	Integrative treatment	24	8
Chen et al. (23)	2022	46	50	4.20 ± 1.10	3.90 ± 0.80	26/20	28/22	MT (Mixed methods) + rehabilitation treatment	Rehabilitation treatment	24	8
Hu et al. (24)	2021	25	25	4.80 ± 1.30	4.90 ± 1.40	14/11	15/10	MT (IMT)+ comprehensive intervention	comprehensive intervention	12	8
Zhao et al. (25)	2020	38	30	8.30 ± 4.00	8.60 ± 4.30	22/16	21/9	MT (Community MT) + routine treatment	Routine treatment	15	8
Li et al. (26)	2020	33	33	3.46 ± 1.02	3.33 ± 0.99	24/9	23/10	MT (IMT)+ floor time therapy	Floor time therapy	24	8
Zhou et al. (27)	2019	37	36	4.35 ± 1.46	5.52 ± 1.02	31/6	26/10	MT (Receptive methods) + integrative therapy	Integrative therapy	8	8
Niu et al. (28)	2019	30	30	4.62 ± 0.25	4.55 ± 0.34	24/6	23/7	MT (Mixed methods) + language training	Language training	8	8
Zhou et al. (29)	2018	48	48	4.13 ± 1.20	3.94 ± 1.02	31/17	32/16	MT (Mixed methods) + general rehabilitation training	General rehabilitation training	10	7
Tian et al. (30)	2018	43	43	3.51 ± 1.38	3.23 ± 1.32	25/18	23/20	MT (Community MT) + analytical therapy	Analytical therapy	12	7
Li et al. (31)	2018	47	47	5.94 ± 1.38	6.31 ± 1.52	26/21	28/19	MT (IMT)+ routine rehabilitation training	Routine rehabilitation training	48	7
Nie et al. (32)	2017	35	35	2.20 ± 0.80	2.40 ± 0.70	28/7	26/9	MT (Mixed methods) + language training	Language training	24	8
Huang et al. (33)	2015	30	30	5.10 ± 1.30	4.70 ± 1.20	22/8	25/5	MT (Mixed methods) + medical education	Medical Education	8	8

M/F, male versus female; MT, music therapy; IMT, Improvisational MT; NA, not available.

### The effect of music therapy on ATEC score

**Figure 2:**
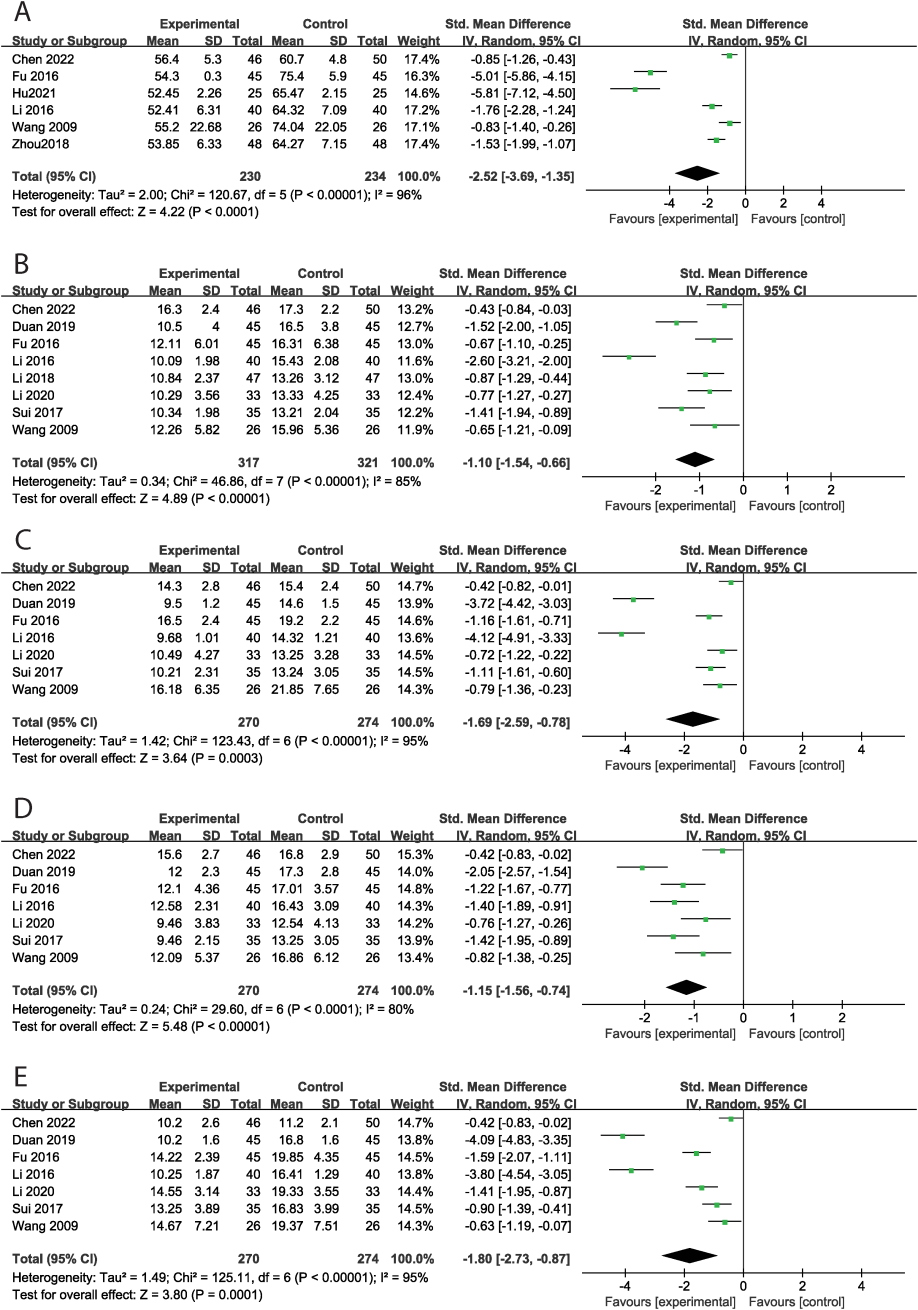
The effect of music therapy on ATEC score [**(A)** total ATEC score, **(B)** communication skills, **(C)** social interaction skills, **(D)** language ability, **(E)** cognitive function].

Six studies reported the total ATEC score, encompassing 464 cases, with 230 in the experimental group and 234 in the control group. MT significantly decreased the total ATEC score (SMD = -2.52, 95% CI: -3.69 ~ -1.35, P < 0.001) compared to the control group, with high levels of heterogeneity across studies (Tau² = 2.00, Chi² = 120.67, I² = 96%, P < 0.001) (**Figure 2A**). Subgroup analyses by intervention duration and modality were conducted to explore heterogeneity sources. MT significantly reduced total ATEC scores in both the >12-week subgroup (SMD = -2.20, P < 0.001; I² = 97.5%, P < 0.001) and the ≤12-week subgroup (SMD = -2.87, P < 0.001; I² = 94.6%, P < 0.001). Similarly, high heterogeneity accompanied the significant benefits observed in both MT + rehabilitation training and MT + language training subgroups. Specifically, eight studies evaluated communication skills, finding that MT significantly improved these skills (SMD = -1.10, 95% CI: -1.54 ~ -0.66, P < 0.001) compared to the control group, with considerable heterogeneity among studies (Tau² = 0.34, Chi² = 46.86, I² = 85%, P < 0.001) (**Figure 2B**). Seven studies assessed social interaction skills, three of which utilized a subscale of the ATEC. MT significantly improved social interaction skills (SMD = -1.69, 95% CI: -2.59 ~ -0.78, P < 0.001) compared to the control group, with high levels of heterogeneity (Tau² = 1.42, Chi² = 123.43, I² = 95%, P < 0.001) (**Figure 2C**). Additionally, seven studies examined language ability. MT significantly enhanced language ability (SMD = -1.15, 95% CI: -1.56 ~ -0.74, P < 0.001) compared to the control group, with high heterogeneity across studies (Tau² = 0.24, Chi² = 29.60, I² = 80%, P < 0.001) (**Figure 2D**). Furthermore, seven studies assessed cognitive function, three of which employed a subscale of the ATEC. MT significantly improved cognitive function (SMD = -1.80, 95% CI: -2.73 ~ -0.87, P < 0.001) compared to the control group, with substantial heterogeneity across studies (Tau² = 1.49, Chi² = 125.11, I² = 95%, P < 0.001) (**Figure 2E**). Funnel plot asymmetry did not reveal any potential for publication bias (P>0.05) (**Supplementary Figure S1**).

### The effect of music therapy on ABC score

**Figure 3:**
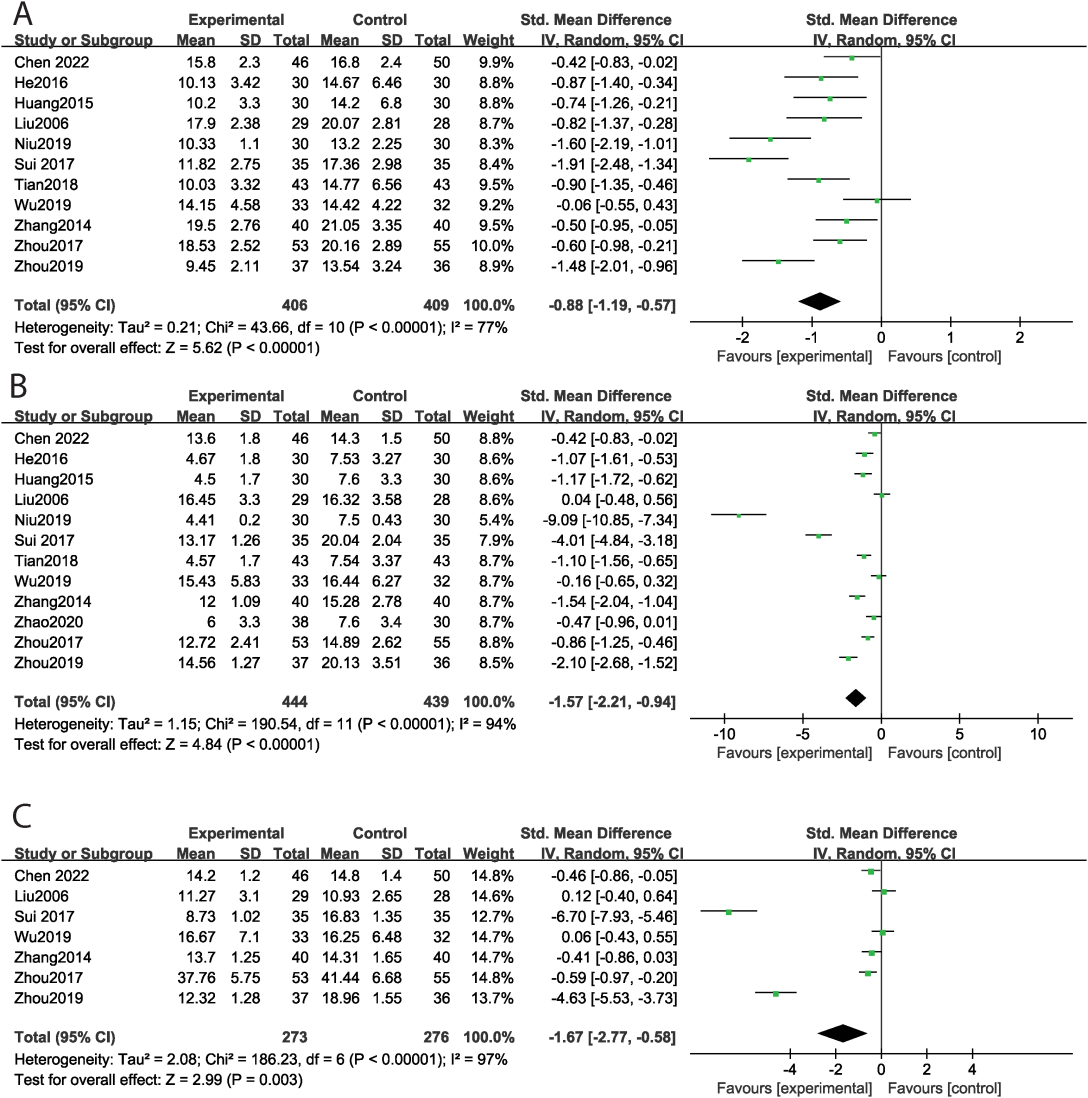
The effect of music therapy on ABC score [**(A)** total ABC score, **(B)** sensory, **(C)** social and self-help].

**Figure 4:**
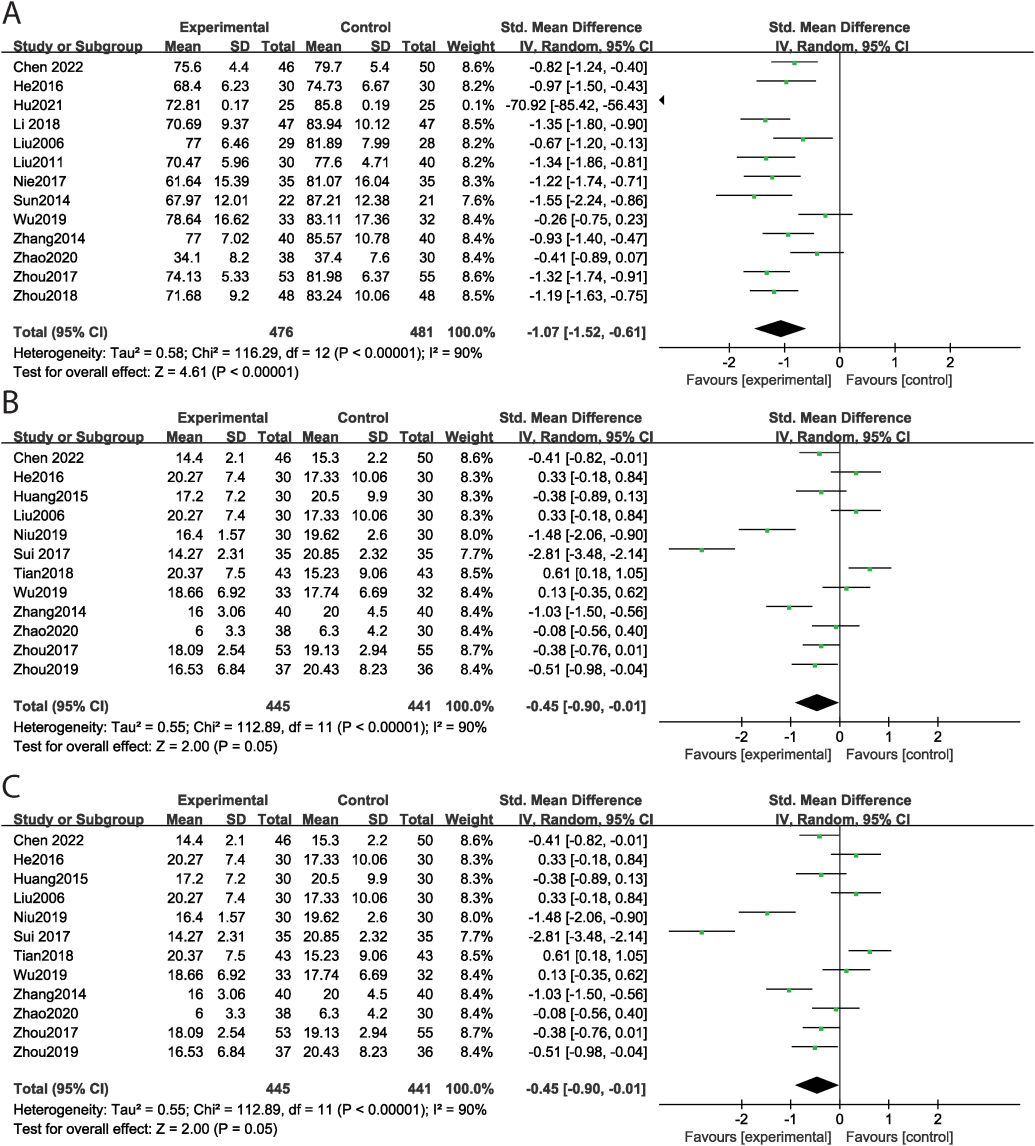
The effect of music therapy on ABC score [**(A)** body and object use, **(B)** language skills, **(C)** relating].

Thirteen studies reported the total ABC score, encompassing 815 cases, with 406 in the experimental group and 409 in the control group. MT significantly reduced the total ABC score (SMD = -1.07, 95% CI: -1.52 ~ -0.61, P < 0.001) compared to the control group, with high levels of heterogeneity observed across studies (Tau² = 0.58, Chi² = 116.29, I² = 90%, P < 0.001) (**Figure 3A**). Subgroup analyses by intervention duration were conducted to explore heterogeneity sources. MT significantly reduced total ABC scores in both the >12-week subgroup (SMD = -0.87, P < 0.001; I² = 71.0%, P = 0.002) and the ≤12-week subgroup (SMD = -1.58, P < 0.001; I² = 94.6%, P < 0.001). Specifically, eleven studies focused on the sensory dimension. MT significantly improved sensory outcomes (SMD = -0.84, 95% CI: -1.25 ~ -0.44, P < 0.001) compared to the control group, again with high levels of heterogeneity across studies (Tau² = 0.41, Chi² = 76.91, I² = 87%, P < 0.001) (**Figure 3B**). Twelve studies evaluated the social and self-help dimensions, finding that MT significantly improved these areas (SMD = -0.45, 95% CI: -0.90 ~ -0.01, P = 0.05), despite high heterogeneity across studies (Tau² = 0.55, Chi² = 112.89, I² = 90%, P < 0.001) (**Figure 3C**). Eleven studies assessed body and object use, showing a significant improvement with MT (SMD = -0.88, 95% CI: -1.19 ~ -0.57, P < 0.001), along with high levels of heterogeneity (Tau² = 0.21, Chi² = 43.66, I² = 77%, P < 0.001) (**Figure 4A**). In terms of language skills, eleven studies also reported significant improvements due to MT (SMD = -1.57, 95% CI: -2.21 ~ -0.94, P < 0.001), with high heterogeneity across studies (Tau² = 1.15, Chi² = 190.54, I² = 94%, P < 0.001) (**Figure 4B**). Furthermore, seven studies assessed the relating dimension; however, no significant difference was found between the experimental and control groups (SMD = -1.67, 95% CI: -2.77 ~ -0.58, P = 0.003), with high levels of heterogeneity evident (Tau² = 2.08, Chi² = 186.23, I² = 97%, P < 0.001) (**Figure 4C**). Funnel plot asymmetry did not reveal any potential for publication bias (P>0.05) (**Supplementary Figure S2**).

### The effect of music therapy on CARS score

**Figure 5:**
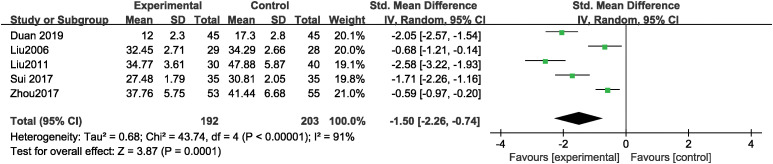
The effect of music therapy on CARS score.

Five studies reported the total CARS score, containing 815 cases with 192 cases in the experimental group and 203 in the control group. MT significantly decreased total CARS score (SMD = -1.50, 95% CI: -2.26 ~ -0.74, P < 0.001) compared to the control group, with high levels of heterogeneity across studies (Tau² = 0.68, Chi² = 43.74, I² = 91%, P < 0.001) (**Figure 5**). Subgroup analyses by intervention duration were conducted to explore heterogeneity sources. MT significantly reduced total CARS scores in both the >12-week subgroup (SMD = -2.09, P < 0.001; I² = 49.9%, P = 0.136) and the ≤12-week subgroup (SMD = -0.62, P < 0.001; I² = 0%, P=0.788). Weights were calculated by the inverse‐variance method, with each study’s contribution proportional to the inverse of its effect-estimate variance. 14 carries the highest weight (21.0%), reflecting its larger sample size more precise estimates are given more influence in the overall effect size calculation. Leave-one-out sensitivity analysis demonstrated that the total results remained unchanged after excluding each study separately. Funnel plot asymmetry did not reveal any potential for publication bias (P>0.05) (**Supplementary Figure S3**).

## Discussion

The aim of this meta-analysis was to estimate the effectiveness of music therapy (MT) for children with Autism Spectrum Disorder (ASD) in the Chinese population. Our review of twenty-three studies showed that MT was effective in reducing autism-related symptoms as assessed by various standardized tools. Specifically, MT demonstrated significant improvements in key areas such as communication, social interaction, language skills, and cognitive function when compared to control groups. The significant reduction in ATEC scores suggests that MT effectively improves core autism symptoms, including communication, social interaction, and behavioral functioning, reinforcing its potential as a valuable intervention for addressing the multifaceted challenges of ASD. The concurrent decrease in ABC scores further supports MT’s role in reducing maladaptive behaviors, though the comparatively smaller effect implies a stronger impact on socio-communicative domains than behavioral regulation alone. These findings highlight the promising role of MT as an intervention that can enhance various aspects of functioning in children with ASD.

The science of MT represents a multifaceted interdisciplinary field that combines psychology, medicine, music, and education, highlighting music’s vital role in healing and well-being (34). Music’s complexity arises from its ability to engage multiple areas of the brain simultaneously, leading to various proposed mechanisms for its effects. One such mechanism is neuroplasticity, which refers to the brain’s capacity to form new connections and its association with feelings of reward(T. 35). Another important principle is Hebbian theory, which suggests that when two neurons fire together, they are more likely to establish a new connection (36). Key concepts in MT include neuroendocrine theory, resonance theory, psychological mechanisms, and the energy spectrum of music wave theory (37, 38). The acoustic properties of music interact with the limbic system and the reticular formation in the brainstem, enhancing nerve cell excitability. Through rhythm and melody, music produces complex auditory stimuli that can evoke emotional responses, enriching therapeutic experiences. MT is particularly beneficial for promoting brain development in children, aiding in the enhancement of various skills such as attention, memory, imagination, abstract thinking, and language (39, 40). Within the therapeutic framework, musical and emotional attunement fosters synchronization, supports sensory integration and emotional regulation, and enables the sharing of feelings, leading to the creation of a shared narrative among participants (41).

Music interventions can significantly enhance the engagement of children with ASD in learning and social activities. Activities such as singing, playing instruments, rhythm training, music games, and listening to music serve to rebuild, sustain, and promote both mental and physical health while simultaneously fostering language and social skills, improving mood, and enhancing cognitive abilities in children with ASD (42). These findings align with the research conducted by Ghasemtabar et al. (43), which demonstrated an increase in social skills among children who received MT. Similarly, the study by Vaiouli et al. (44) showed improvements in social skills through improvised MT. When implementing MT with children with ASD, careful consideration should be given to the selection of music. Appropriate compositions and rhythms should be chosen based on the child’s age and specific needs, and a blend of group therapy with individualized approaches is often beneficial. The physical movements and musical interactions in MT parallel the musical exchanges typically seen in early childhood, facilitating spontaneous moments of attunement and bodily synchronization (45). Moreover, various elements of music have a profound impact on human physiology; for instance, the tempo of a musical piece can influence cardiovascular responses, while harmonic consonance activates areas of the paralimbic system and cortex, whereas dissonance can evoke feelings of roughness (46). Children who experience disorganized sensory perception, restricted interests, or heightened emotional responses may particularly benefit from methods that focus on sensory integration and affect regulation, laying the foundation for the development of cross-modal perception, body coherence, and self-regulation-key intrapersonal components critical for developing intersubjectivity (47, 48). Thus, greater emphasis should be placed on the bodily-affective regulation of each child during MT sessions. Musical rhythm training challenges children to encode, maintain and update temporal sequences in real time, repeatedly engaging fronto-parietal circuits that underlie working memory (49). Sustained entrainment drives plasticity in executive pathways, enhancing the capacity to hold and manipulate information (50, 51). As working memory improves, children also gain in attention control, language processing and problem-solving, accounting for the robust cognitive benefits of music-based interventions (52, 53).

Recent fMRI research indicates that musical stimulation can simultaneously strengthen under-connected intrinsic networks and dampen hyper-reactive limbic responses in children with ASD. For instance, exposure to structured musical excerpts has been shown to increase functional connectivity among core hubs of the default mode network, such as fronto-temporal brain networks, and auditory and subcortical regions (54, 55). Emotional music also activates the parahippocampal gyrus extending into the amygdala regardless of whether it is happy or sad, and engages the thalamus and adjacent midbrain regions associated with arousal and the processing of temporal complexity (56, 57). By engaging both large-scale social networks and conserved emotional arousal circuits, music therapy may drive the social interaction, communication, and emotional regulation improvements seen in ASD.

Several limitations exist in our study. Firstly, only articles published in English and Chinese were considered, which may introduce publication bias. Additionally, there has been a decline in the number of published studies in recent years, suggesting the possibility of selective publication. Some trials combined music therapy with other interventions, such as language training, making it difficult to isolate the effects of music therapy alone. The broad age range across studies limits our ability to draw conclusions about age-specific efficacy. Most included studies did not report the music therapists’ credentials or training levels, limiting our ability to assess the influence of therapist expertise on intervention outcomes. The primary outcomes assessed varied significantly among studies, and the lack of consistent measures of interaction further adds to the heterogeneity observed. To establish the effectiveness of MT for children with ASD, future research needs to feature large sample sizes, employ rigorous methodologies, and implement long-term observation and follow-up periods. Furthermore, adopting standardized research designs is essential for drawing more robust conclusions regarding the benefits of MT in the treatment of children with ASD.

## Conclusions

The results of the meta-analysis indicate that music therapy significantly enhances the overall symptoms of autism spectrum disorder in children from the Chinese population, particularly by improving communication skills, social interaction, language abilities, and cognitive function within existing high-quality studies. These robust findings support the integration of structured music therapy into pediatric rehabilitation protocols. However, the heterogeneity among the studies included and the low methodological quality of some research underscore the necessity for further well-designed randomized controlled trials with larger sample sizes to confirm and solidify these findings.
