# Supplementary material for: Effect of music therapy on children with autism spectrum disorders in the Chinese population: a systematic review and meta-analysis
Source: Front Psychiatry. 2025 Jul 25;16:1611182. doi: 10.3389/fpsyt.2025.1611182 (PMC12332751; doi:10.3389/fpsyt.2025.1611182)
Supplement: Supplementary file 1 [file DataSheet1.docx]

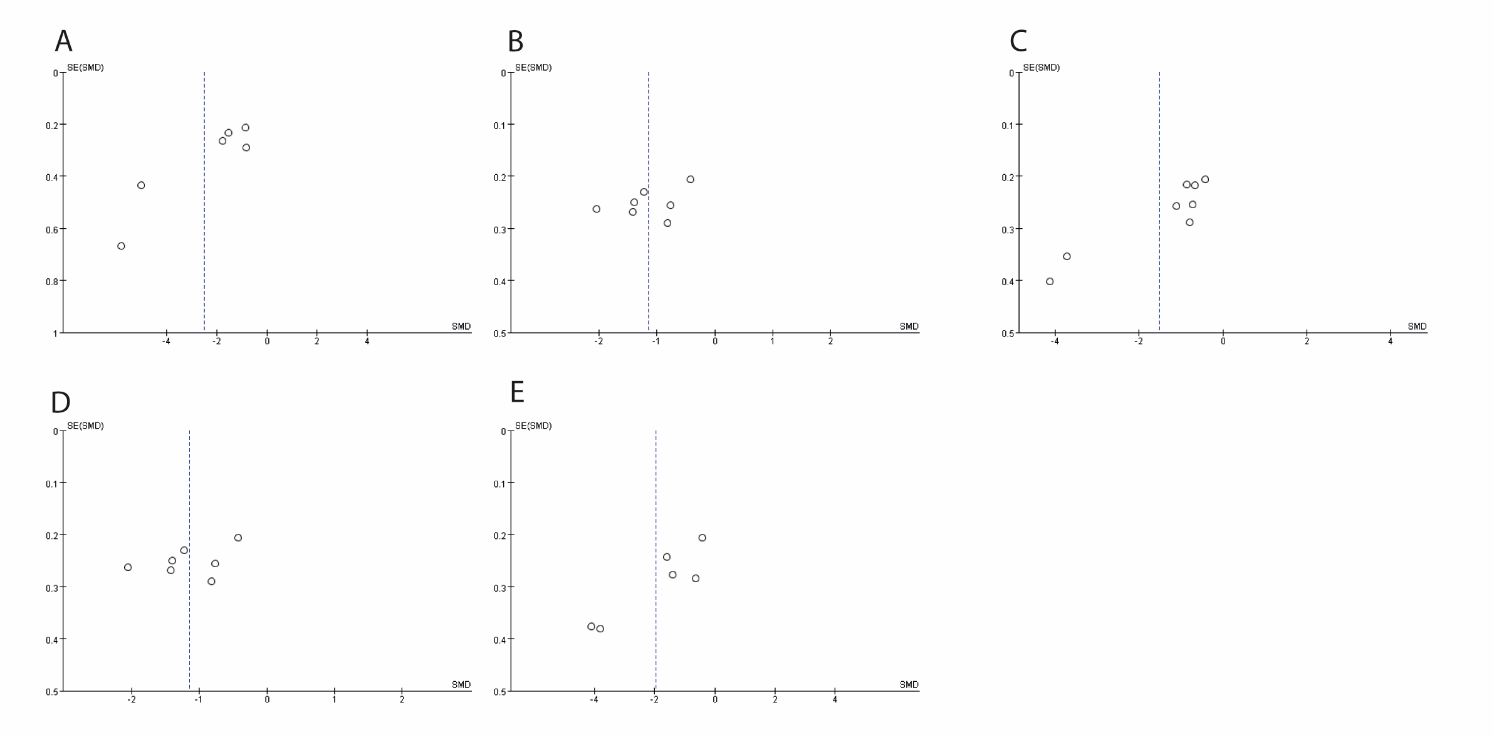


Figure S1. A funnel plot assesses the risk of publication bias in the effect of music therapy on the ATEC score (A: total ATEC score, B: communication skills, C: social interaction skills, D: language ability, E: cognitive function).


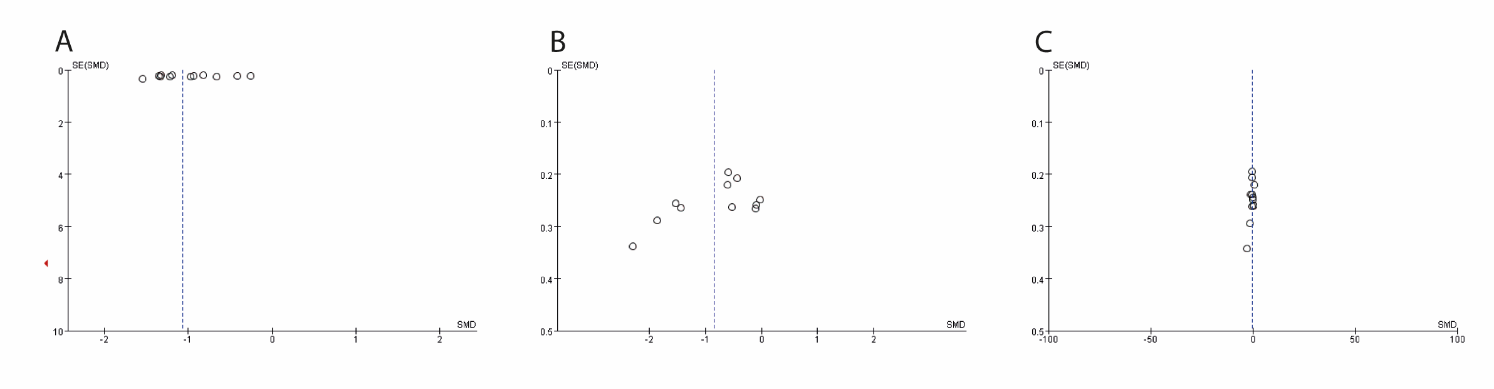


Figure S2. A funnel plot assesses the risk of publication bias in the effect of music therapy on the ABC score (A: total ABC score, B: sensory, C: social and self-help).


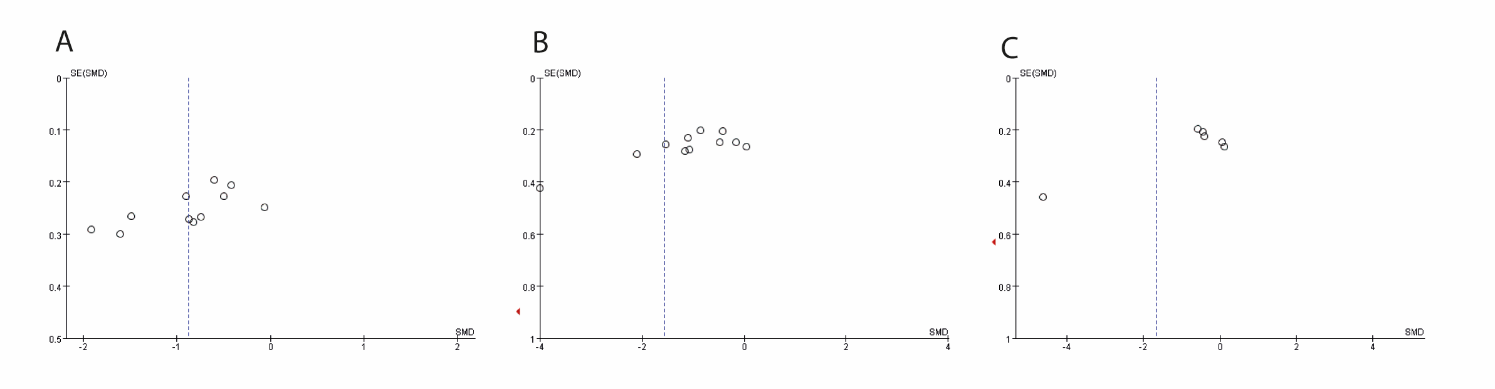


Figure S3. A funnel plot assesses the risk of publication bias in the effect of music therapy on the ATEC score (A: body and object use, B: language skills, C: relating).


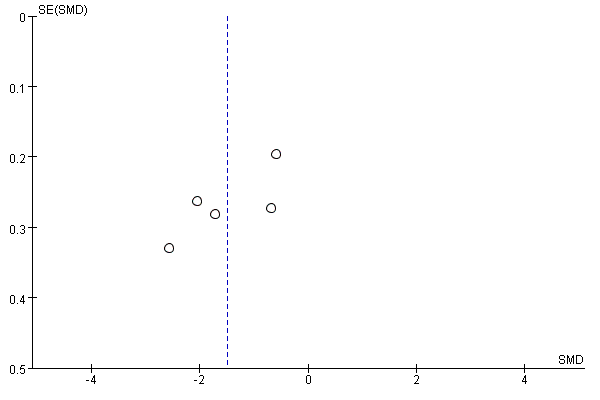


Figure S4. A funnel plot assesses the risk of publication bias in the effect of music therapy on the CARS score.
